# Comparison between Random Forests, Artificial Neural Networks and Gradient Boosted Machines Methods of On-Line Vis-NIR Spectroscopy Measurements of Soil Total Nitrogen and Total Carbon

**DOI:** 10.3390/s17102428

**Published:** 2017-10-24

**Authors:** Said Nawar, Abdul M. Mouazen

**Affiliations:** 1 Department of Soil Management, Ghent University, Coupure 653, 9000 Gent, Belgium; abdul.mouazen@ugent.be; 2 Cranfield Soil and AgriFood Institute, School of Water, Energy and Environment, Cranfield University, Cranfield MK43 0AL, UK; 3 Faculty of Agriculture, Suez Canal University, Ismailia 41522, Egypt

**Keywords:** on-line vis-NIR measurement, total nitrogen, total carbon, spiking, gradient boosted machines, artificial neural networks, random forests

## Abstract

Accurate and detailed spatial soil information about within-field variability is essential for variable-rate applications of farm resources. Soil total nitrogen (TN) and total carbon (TC) are important fertility parameters that can be measured with on-line (mobile) visible and near infrared (vis-NIR) spectroscopy. This study compares the performance of local farm scale calibrations with those based on the spiking of selected local samples from both fields into an European dataset for TN and TC estimation using three modelling techniques, namely gradient boosted machines (GBM), artificial neural networks (ANNs) and random forests (RF). The on-line measurements were carried out using a mobile, fiber type, vis-NIR spectrophotometer (305–2200 nm) (AgroSpec from tec5, Germany), during which soil spectra were recorded in diffuse reflectance mode from two fields in the UK. After spectra pre-processing, the entire datasets were then divided into calibration (75%) and prediction (25%) sets, and calibration models for TN and TC were developed using GBM, ANN and RF with leave-one-out cross-validation. Results of cross-validation showed that the effect of spiking of local samples collected from a field into an European dataset when combined with RF has resulted in the highest coefficients of determination (R^2^) values of 0.97 and 0.98, the lowest root mean square error (RMSE) of 0.01% and 0.10%, and the highest residual prediction deviations (RPD) of 5.58 and 7.54, for TN and TC, respectively. Results for laboratory and on-line predictions generally followed the same trend as for cross-validation in one field, where the spiked European dataset-based RF calibration models outperformed the corresponding GBM and ANN models. In the second field ANN has replaced RF in being the best performing. However, the local field calibrations provided lower R^2^ and RPD in most cases. Therefore, from a cost-effective point of view, it is recommended to adopt the spiked European dataset-based RF/ANN calibration models for successful prediction of TN and TC under on-line measurement conditions.

## 1. Introduction

Estimation of carbon and nitrogen status in the soil is crucial from both agricultural and environmental points of view. It is well known that soil total carbon (TC) and total nitrogen (TN) are vital factors for soil fertility and crop production [1,2]. Traditional laboratory analysis methods for TN and TC are laborious, time-consuming, costly and destructive [3,4]. Therefore, proximal soil sensing (PSS) techniques, in particular visible and near infrared (vis-NIR) reflectance spectroscopy can be considered as a cost-effective and alternative technique for estimating TN and TC [5,6].

On-line (tractor-driven) vis-NIR spectroscopy offers the possibility of collecting high spatial resolution data, compared with conventional laboratory analyses. However, on-line spectroscopic measurements are affected by ambient and experimental conditions that need to be overcome for accurate prediction to be achieved. One way to reduce these negative influences is by adopting advanced multivariate calibrations techniques, particularly those approaches that account for nonlinearity between NIR spectral response and soil properties [5]. Furthermore, overlapping of absorption bands of those properties and scatter effects result in complex absorption patterns, which cannot be derived using simple correlation or linear techniques [7].

Non-linear regression has been introduced in the literature as the best option to model spectroscopic data [8,9]. Among those models, support vector machines (SVM) [5,9], artificial neural networks (ANNs) [10,11], boosted regression trees [12], multivariate adaptive regression splines (MARS) [9,13] and random forests (RF) [14,15] were proven to provide improved prediction performances as compared to the linear partial least squares regression (PLSR) for modelling nonlinear phenomena like soil properties [8,11]. Neural networks, specifically multilayer perceptrons (MLPS), are mathematical models that use learning algorithms inspired by the brain to store information [16]. They have been examined in the field of spectroscopy using simulated data [17]. They have been used successfully to model a complex spectral library including over 1100 soil samples for large-scale study [14], and were used to predict OC based on on-line vis-NIR measurements, outperforming PLSR with ratio of prediction deviation (RPD) of and 2.28 [11]. However, overfitting is a major problem for ANN analysis, which has required special data pretreatment [18].

Recently, RF has received growing attention in vis-NIR spectral analyses in different domains. It is an ensemble learning technique, introduced by Breiman [19], as a combination of tree predictors that is robust and rarely overfits; it hence yields highly accurate predictions [19,20,21]. Accordingly, RF can handle nonlinear and hierarchical behaviors when introducing variability to the general spectral library for predicting local samples. Boosting trees (BT) characterized by the stochastic that enhances predictive performance, decreases the variance of the final model, by utilizing only a arbitrary subset of data to match each new tree [22]. Viscarra Rossel and Behrens [14] have applied BT to predict soil OC, pH and clay content using non-mobile (laboratory-based) spectroscopy measurement. Gradient boosted machines (GBM) is a hybrid method that incorporates both boosting and bagging approaches [22,23]. It performs boosting through choosing, at each step, the arbitrary sample of the data ultimately causing a progressive enhancement of the model performance [23]. GBM has been used successfully in digital mapping of OC [23,24,25]. Despite the importance of RF and GBM, no study on the use of both modelling methods for on-line spectroscopy measurement of soil properties can be found in the literature. The hypothesis of this study is that both GBM and RF outperform ANN for the on-line prediction of soil TN and TC.

The main goal of this paper is to compare the performance of GBM, ANNs and RF for the on-line prediction of TN and TC based on local (single field) dataset from two target fields and spiking of local samples of these two target fields into an European dataset.

## 2. Materials and Methods

### 2.1. Experimental Sites

Two experimental fields were used in this study, namely, Hessleskew and Hagg with total area of about 12 ha and 21 ha, respectively, both located in Yorkshire (Hessleskew, longitudes −0.590° and −0.586° W, and latitudes 53.844° and 53.844° N; Hagg, longitudes of −1.172° and −1.166° W, and latitudes of 53.936° and 53.941° N), The United Kingdom. Hessleskew field is cultivated with cereal crops in rotation, where Hagg field is cultivated with vegetable crops (e.g., carrots, cabbage, onions and leeks). The soil texture for the Hessleskew and Hagg fields is clay and sandy loam, respectively, according to United States Department of Agriculture (USDA) textural soil classification system [26].

### 2.2. On-Line Soil Measurement and Collection of Soil Samples

**Figure 1:**
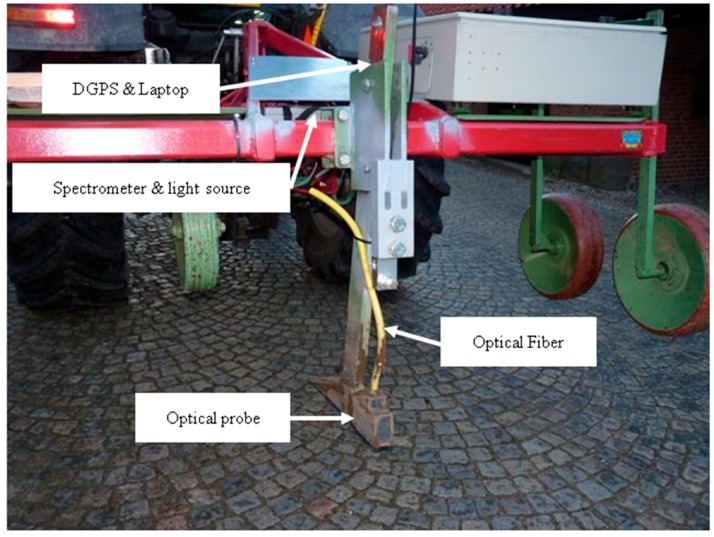
The on-line visible and near infrared (vis-NIR) spectroscopy sensor developed by Mouazen [27].

Both fields were scanned using the on-line system designed and developed by Mouazen [27]. This is a multi-sensor platform consists of a subsoiler, which penetrates the soil to any depth (5–50 cm), creating a trench, whoever bottom part is smoothened with the downwards forces acting on the subsoiler. The subsoiler has been retrofitted with the optical probe and attached to a frame. It was installed into the three point hitch of the tractor. The optical measurement was performed using an AgroSpec mobile, fibre type, vis-NIR spectrophotometer (Tec5 Technology for Spectroscopy, Geramany) with spectral range of 305–2200 nm. A differential global positioning system (DGPS) (EZ-Guide 250, Trimble, Sunnyvale, CA, USA) was utilized to record the positioning associated with on-line measured spectra along with sub-meter precision (Figure 1). The on-line measurement had been completed after previous crop harvest in summer of 2015 and 2016 for Hessleslekew and Hagg fields, respectively. The subsoiler was dragged at parallel transects of 12 m apart, setting the subsoiler tip at about 15 cm deep. A total of 122 and 149 soil samples were collected during the on-line measurement from the former and latter fields, respectively. These samples were used for calibration and validation of the vis-NIR sensor.

### 2.3. Laboratory Chemical and Optical Measurements

Fresh soil samples were used in the laboratory spectral and chemical analyses. Each soil sample was placed in a glass container and mixed well then divided into two parts. The first part was used to fill three Petri dishes of 2 cm in diameter 2 cm deep, representing three replicated measurements. Each soil sample were packed into plastic Petri dishes for soil scanning using the same spectrometer used in the on-line measurements. To obtain optimal diffuse reflection, and hence a good signal-to-noise ratio, all plant and pebble particles were manually removed and the surface was pressed gently with a spatula to be smooth before scanning. A total of ten scans were collected from each replicate, and these were averaged into one spectrum. The second part of each sample was air dried before it was analyzed for total carbon (TC) using the combustion method. This was done by oxidizing the carbon to carbon dioxide (CO_2_) by heating the soil to at least 900 °C on a flow of oxygen-containing gas that is free from carbon dioxide. The amount of carbon dioxide released is then measured by a thermal conductivity detector (TCD). When the soil is heated to a temperature of at least 900 °C, any carbonates present are completely decomposed [28]. The total nitrogen was determined using the Dumas method by heating the soil to a temperature of at least 900 °C in the presence of oxygen gas. During oxidized combustion, mineral and organic nitrogen compounds produce the oxidation products NOx, in addition to molecular nitrogen (N_2_). Copper in the reduction tube quantitatively reduces these nitrogen oxides to N_2_ and binds excess oxygen. The amount of nitrogen is then measured by a TCD [29].

### 2.4. Spectra Pretreatment

The raw average spectra of the on-line and laboratory scanning were subjected to pre-processing, including successively, noise cut, maximum normalization, first derivative and smoothing using the prospectr-R package [30]. First, the spectral range outside 370–1979 nm was cut to remove the noise at both edges. Then, a moving average with five successive wavelengths was used to reduce noise. Maximum normalization followed, which is typically used to get all data to approximately the same scale, with maximum values of 1. The maximum normalisation led to better results for the measurement of TC and TN as compared to the other pre-treatment options tested, including mean and peak normalization. Spectra were then subjected to first derivation using gap–segment derivative (gapDer) algorithms [31] with a second-order polynomial approximation. This method enables the first or higher-order derivatives, including a smoothing factor, to be computed, which determines how many adjacent variables will be used to estimate the polynomial approximation used for derivatives. This gapDer resulted in a better performance than second derivative that increased the noise and reduced the quality of models’ prediction performance. Finally, smoothening with the Savitzky–Golay technique was carried out to remove noise from the spectra.

### 2.5. Dataset Set Selection and Modelling Techniques

The following two data sets were considered in this study:

1. Local dataset: where samples collected from two fields (Hessleskew, *n* = 122; Hagg, *n* = 149),
2. European dataset (*n* = 528), where a total of 528 samples collected from five European countries, namely, Germany (150 samples from two fields), Denmark (147 samples from five fields), the Netherlands (43 samples from one field), Czech Republic (99 samples from four fields), and the UK (89 samples from four fields) were collected.

The Kennard–Stone algorithm [32] was used to select the calibration set (75%), and the rest of the samples (25%) were assigned for the prediction set. The Kennard–Stone algorithm allows to select points (samples) with a uniform distribution over the predictor space. It begins through selecting the pair of samples that are the farthest apart. They are assigned to the calibration set and removed from the dataset. Then, the procedure assigns remaining samples to the calibration set by computing the distance between each unassigned samples *i*_0_ and selected samples *i* and finding the sample *i*_0_ for which:

(1) $$d_{selected}=\underset{i_{0}}{max}(\underset{i}{min}(d_{i,\;i_{0}}))$$

This essentially selects sample *i*_0_, which is the farthest apart from its closest neighbors *i* in the calibration set based on the the Mahalanobis distance (H), which can be defined as the dissimilarity measure matrix (H) between samples in a given matrix X and can be computed as follows:

(2) $$H\left(x_{i\;},x_{j\;}\right)=\sqrt{{\left(x_{i\;},x_{j\;}\right)}^{M-1}{\left(x_{i\;},x_{j\;}\right)}^{T}}$$

where M is the variance–covariance matrix and vector T. The algorithm uses the H that can be achieved by performing a PCA analysis on the input data and computing H as follows:

(3) $$H_{ij}^{2}=\overset{A}{\underset{a=1}{\sum }}{\left({\hat{t}}_{ia}-{\hat{t}}_{ja}\right)}^{2}/{\hat{\lambda }}_{ia}$$

where ${\hat{t}}_{ia}$ is the a^th^ principal component score of sample *i*, ${\hat{t}}_{ja}$ is the corresponding value for sample *j*, ${\hat{\lambda }}_{ia}$ is the eigenvalue of principal component a, and A is the number of principal components included in the computation [30].

Spiking was used to introduce the local variability of the two experimental fields into the European dataset. A total of 85 and 110 samples that have been selected from the Hessleskew and Hagg fields, respectively, using the Kennard–Stone algorithm were spiked into the European dataset.

Before running the analysis, the entire dataset of each target field (Hesselskew or Hagg) was divided into 75% for calibration, and 25% for prediction as described above. This was done for both the laboratory and on-line collected soil spectra. The 75% soil samples were used for developing the calibration models for the local dataset (single field), and the spiked European dataset model. To evaluate these models, cross-validation technique with leave-one-out cross-validation (LOOV) was performed on the training data (75%). For independent validation, the laboratory reference measurement values of the prediction set (25%), e.g., 37 and 39 samples from Hessleskew and Hagg, respectively, were compared with the laboratory and on-line predicted concentration values at the same positions.

#### 2.5.1. Random Forests Regression

Random forests (RF) is an ensemble learning method developed by Breiman [19], which can be described as follows:

Suppose we have a calibration set $C=\left\{C_{1},\;\ldots .,\;C_{n}\right\}$ with $C_{i}\equiv \left(x_{i},\;y_{i}\right)$ and an independent test case $C_{0}$ with predictor $x_{0}$, the following steps can be carried out:

(1) Sample the calibration set C with replacement to generate bootstrap resamples $B_{1},\ldots \;,\;B_{M}$
(2) For each resample $B_{m},\;m=1,\;\ldots ,\;M$, grow a regression tree $T_{m}$.
(3) For predicting the test case $C_{0}$ with covariate $x_{0}$, the predicted value by the whole RF is obtained by combining the results given by individual trees. Let $\hat{f}{}_{m}^{*}\left(x_{0}\right)$ denote the prediction of $C_{0}$ by mth tree, the RF prediction for regression problems can then be written [33] as:
  (1a) $$\frac{1}{M}\sum_{m=1}^{M}\hat{f}{}_{m}^{*}\left(x_{0}\right)$$

**Figure 2:**
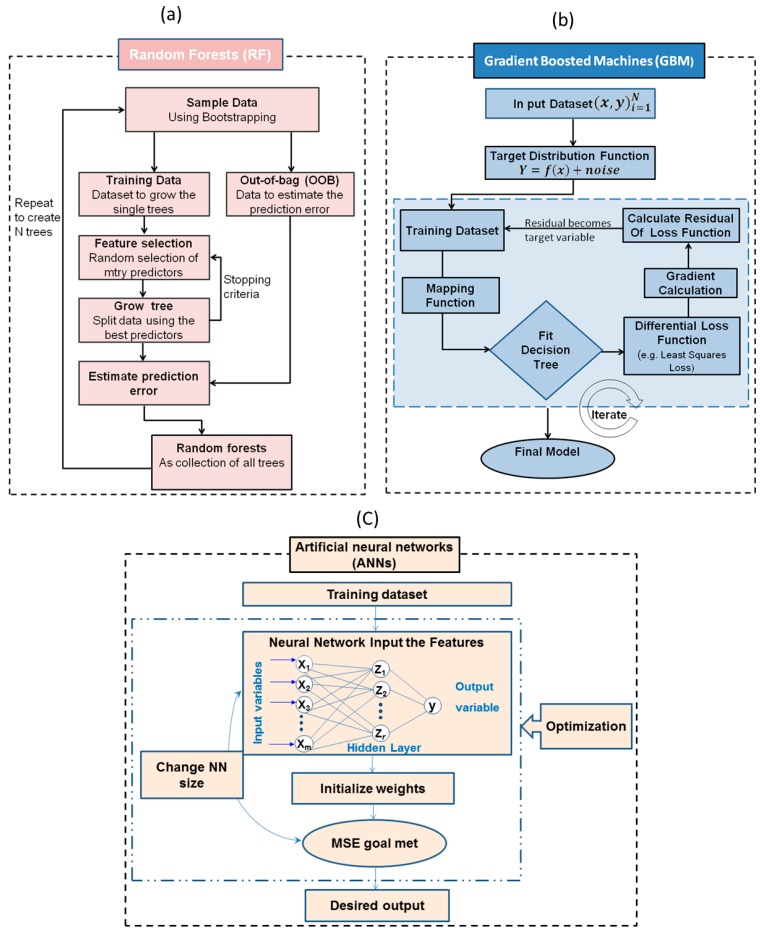
Overview flowchart listing the main steps of the multivariate analysis methods. (**a**) Random forests regression; (**b**) gradient boosted machines (GBM); and (**c**) artificial neural networks (ANNs).

RF is generally used for data classification and regression. The algorithm works by growing an ensemble of regression trees based on binary recursive partitioning, where the algorithm first begins with a number of bootstrap samples (ntree) from the predictor space (original data) [34]. Each bootstrap sample will then grow regression tree with a modifying operation, in which subsequently a number of the predictors (mtry) are randomly sampled, and the algorithm chooses the best split from among those sampled variables rather than considering all variables. The default mtry value is the square root of the total number of variables [35]. Therefore, the number of trees (ntree) needs to be set sufficiently high. Consequently, RF hardly overfits when more trees are added [19], but produce a limited generalisation error [20,36]. The final prediction can be obtained as the mean value of the individual predictions made by each decision tree. RF does not need complicated data pretreatment and runs very fast when compared to other machines learning algorithms such as ANNs and GBM [37], which is a very important factor in the field of on-line and in situ measurements. Figure 2 shows the main processes of the RF algorithm. In this work, an ntree of 100 and an mtry of 2 were used to develop the TN and TC models. These parameters were determined by the tune RF function implemented in the R software package, named random forest version 4.6–12 [38]. The same split of datasets described above (75% calibration, 25% prediction) were utilised for RF analysis.

#### 2.5.2. Gradient Boosted Machines (GBM)

Boosting is a method based on the idea of combining a set of weak learners and delivers superior predictive performance whose always highly accurate [39]. In GBM, the learning procedure sequentially fits new models to the training data, utilizing suitable techniques (loss function, weak learner and additive model) progressively to increase emphasis on observations modelled poorly through the existing collection of trees. This particular enhancement can be achieved through constructing the new base-learners to become maximally related using the negative gradient of the loss function, linked to the entire ensemble (Figure 2). Boosting draws bootstrap samples of the predictor data, fits a tree, and subtracts the prediction from the original data. The trees tend to be iteratively suited to the residuals and the predictions summed up [40]. Steps to avoid overfitting are essential because the sequential nature of boosting allows trees to be added before the model is entirely overfitted [41]. According to Hastie [38] the GBM algorithm can be described as follows:

(2a) $$1-\;\mathrm{Initialize}\;f_{0}\left(x\right)=\arg\;\min_{\gamma }\sum_{i=1}^{N}L\left(Y_{i},\;\gamma \right).$$

This initializes the optimal constant model, which is just a single terminal node tree.

For m=1 to M:

(a) For i=1,2,…,N compute
  (2b) $$r_{im}=-{\left[\frac{\partial L(Y_{i},\;f\left(x_{i}\right))}{\partial f\left(x_{i}\right)}\right]}_{f=f_{m-1}}$$
  The components of the negative gradient are referred to the generalized residuals $r_{im}$ of the current model on the ith observation evaluated at *f* = *f*_m−1_.
(b) Fit a regression tree to the targets $r_{im}$ giving terminal regions
  $$R_{jm},\;\;j=1,2,\ldots \ldots .J_{m}.$$
(c) For $j=1,2,\ldots .,\;J_{m}\;\mathrm{compute}\;$
  (2c) $$\gamma_{jm}=\arg\;\underset{\gamma }{\min}\underset{x_{i}\epsilon R_{jm}}{\sum }L(Y_{i},\;f_{m-1}\left(x_{i}\right)+\;\gamma )$$

γ parameterizes the split variables and split points at the internal nodes, and the predictions at the terminal nodes. In the gbm package ϵ is shrinkage with default 0.001 that to allow at least for 1000 trees. The best fits the current residuals is added to the expansion at each step as in step (d). This produces *fm*(*x*), and the process is repeated. At each iteration m, one solves for the optimal lose function and add to the current expansion $f_{m-1}\left(x\right)$.

The boosting models were fitted by using the code published by Elith et al. [42], which is based on the package gbm in R software. There is a range of tuning parameters for GBM model; shrinkage reduces the participation of each tree to the final model. Shrinkage setup was recommend to be small enough (0.01–0.001) to allow at least for 1000 trees [42]. Hence, for all procedures, shrinkage was set to the lower end of the recommendations (0.001). The subsampling rate “bag fraction” that specifies the ratio of the data to be used at each iteration was set up to the default (0.50). The number of trees (ntree) is more relevant than for random forest, as gradient boosting overfits if ntree is excessive. Hence, ntree was determined for each individual modeling case. The same split of datasets described above (75% calibration, 25% prediction) were utilised for GBM analysis.

#### 2.5.3. Artificial Neural Networks (ANNs)

ANNs are a machine learning framework that attempts to mimic the learning pattern of natural biological neural networks and are based on their ability to “learn” throughout a training procedure exactly where they’re given inputs and a set of anticipated results. ANNs are a machine learning framework that attempts to mimic the learning pattern of natural biological neural networks and are based on their ability to “learn” throughout a training procedure exactly where they're given inputs and a set of anticipated results. The neural network used in this study was a multilayer perceptron (MLP) neural network. It typically consists of an input layer (i.e., spectral data or principal components), one or more hidden layers, where the real processing is performed via a system of weighted ‘connections’, and an output layer (prediction), where the answer is output (Figure 2). They function by linking the input neurons to output neurons, through the connections (weights). The ANNs algorithm with single layer can be described [43] as follows:

First, *r* different linear combinations of the *x*-variables are built

(3a) $${ѵ}_{j}=a_{0j}+\;a_{1j}x_{1}+\ldots +\;a_{mj}x_{m}\;\mathrm{for}\;j=1,\;\ldots .,\;r$$

and then a nonlinear function s—often the sigmoid function—is applied:

(3b) $$z_{j}=\sigma \left({ѵ}_{j}\right)=\frac{1}{1+\exp\left(-{ѵ}_{j}\right)}\;\mathrm{for}\;j=1,\ldots .,\;r$$

Equations (3a) and (3b) constitute a neuron with several inputs *x* and one output *z*.

The new variables *z_j_* can be used in different ways to produce the final output *y*:

(a) as inputs of a neuron with output *y*, (b) in a linear regression model,

(3c) $$y=b_{0}+\;b_{1}z_{1}+\;b_{2}z_{2}+\ldots +\;b_{r}z_{r}+e$$

and (c) in a nonlinear regression model

(3d) $$y=b_{0}+\;b_{1}f_{1}\left(z_{1}\right)+\;b_{2}f_{2}\left(z_{2}\right)+\ldots +\;b_{r}f_{r}\left(z_{r}\right)+e$$

The most straightforward approach was used to build the ANNs model. This is performed using the training and test sets. Samples in the training data sets were the same as those in the calibration sets used in the RF and GBM analyses, whereas the test sets were the same as the prediction set. Leave-one-out cross-validation was used to avoid over-fitting and to monitor the training error. The input layer has the same number of input nodes to the number of soil samples used in each calibration set. The output layer has one node of TN and TC. The number of nodes in the hidden layer was adjusted during the training from 6 to 20 to get the optimised network structure, which resulted in the lowest training error. The training algorithm was selected as stochastic gradient descent, and the training time was set to 1000 times. Exponential and logistic functions were selected for the hidden and the output layers, respectively. The performance of the resultant models were chosen according to the following evaluation parameters: high R^2^ (both in calibration and prediction), and low root mean square error of prediction (RMSEP). The *caret* package [44] has been used to perform the ANN models in R software [45].

### 2.6. Evaluation of Model Accuracy

Model performance for the prediction of TN and TC were evaluated by means of *R*^2^, *RMSEP* and *RPD*, which can be defined as follows:

(4a) $$RMSE=\;\sqrt{\frac{\sum_{i=1}^{n}{\left(\hat{y_{i}}-y_{i}\right)}^{2}}{n-1}}$$

(4b) $$R^{2}=1-\frac{SS_{error}}{SS_{total}}$$

where the $SS_{total}$ and $SS_{error}$ are the variance of measured values and the sum of squared residuals, respectively:

(4c) $$SS_{total}=\sum_{i=1}^{n}{\left(y_{i}-\bar{y}\right)}^{2}$$

(4d) $$SS_{error}=\sum_{i=1}^{n}{\left(y_{i}-\hat{y_{i}}\right)}^{2}$$

(4e) RPD=SD/RMSE

Viscarra Rossel et al. [46] classified the *RPD* values referring to accuracy of modelling into six classes: excellent (*RPD* > 2.5), very good (*RPD* = 2.5–2.0), good (*RPD* = 2.0–1.8), fair (*RPD* = 1.8–1.4), poor (*RPD* = 1.4–1.0), and very poor model (*RPD* < 1.0). In this study, we adopted this model classification criterion to compare between different calibration models in cross-validation and in laboratory and on-line predictions.

## 3. Results

### 3.1. Laboratory Measured Soil Properties

**Figure 3:**
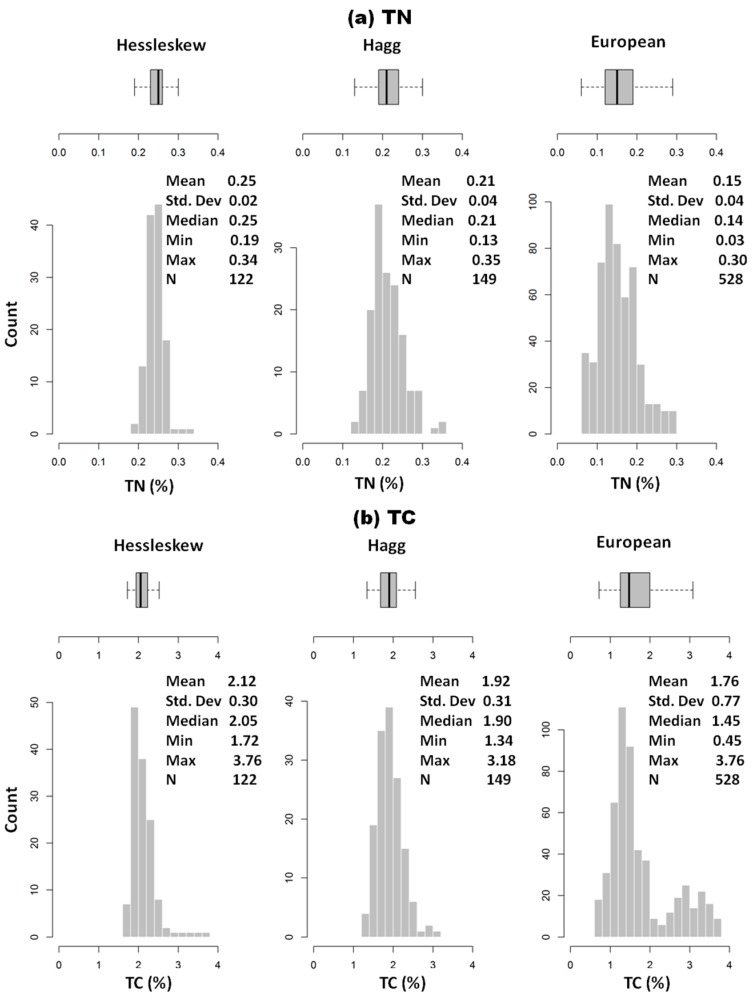
Histograms, box-plots and descriptive statistics of (**a**) soil total nitrogen (TN) and (**b**) total carbon (TC) for Hessleskew and Hagg fields, and European dataset.

The descriptive statistics for measured TN and TC in both fields are shown in Table 1 and Figure 3. It can be observed that TN concentration is low, with mean and maximum values of 0.25% and 0.34%, respectively, whereas the mean and maximum values of TC are 2.12% and 3.67%, respectively, in the Hessleskew field. Both TN and TC in the Hagg field are even smaller than in the Hessleskew field, with mean values of 0.21% and 1.92%, respectively (Table 1). The small range of variability in TN and TC implies these fields are certainly not the optimal case study, as the smaller the variability is, the less successful results can be expected for the prediction capability of the vis-NIR spectroscopy calibration models established [6].

The mean and median of TN are equal in Hessleskew and Hagg, indicating that TN fallows normal distribution, meanwhile European dataset shows left skewed with the mean being greater than the median (0.15 and 0.14%, respectively). The sample distribution of TC is similar to TN in both Hessleskew and Hagg with unimodal as the mean and median values are comparable, whereas the distribution of TC in European dataset shows non-modality and left skewed with the mean being larger than the median (1.67% and 1.45%, respectively).

### 3.2. Performance of the Calibration Models for Predicting TN

**Figure 4:**
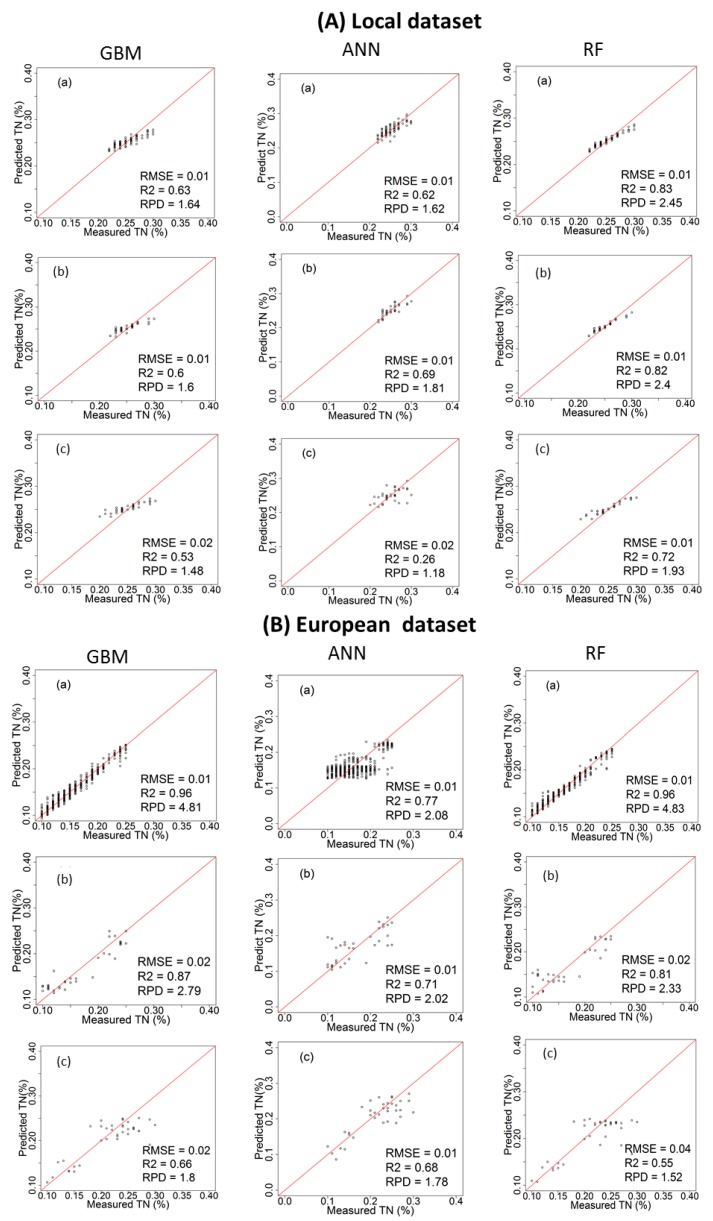
Scatter plots of visible and near infrared (vis-NIR)-predicted versus laboratory-analysed total nitrogen (TN) in Hessleskew field in cross validation (**a**); lab prediction (**b**) and on-line prediction (**c**); using local dataset (**A**) and spiked European dataset (**B**); comparing between gradient boosted machines (GBM), artificial neural network (ANNs) and random forests (RF) models.

**Figure 5:**
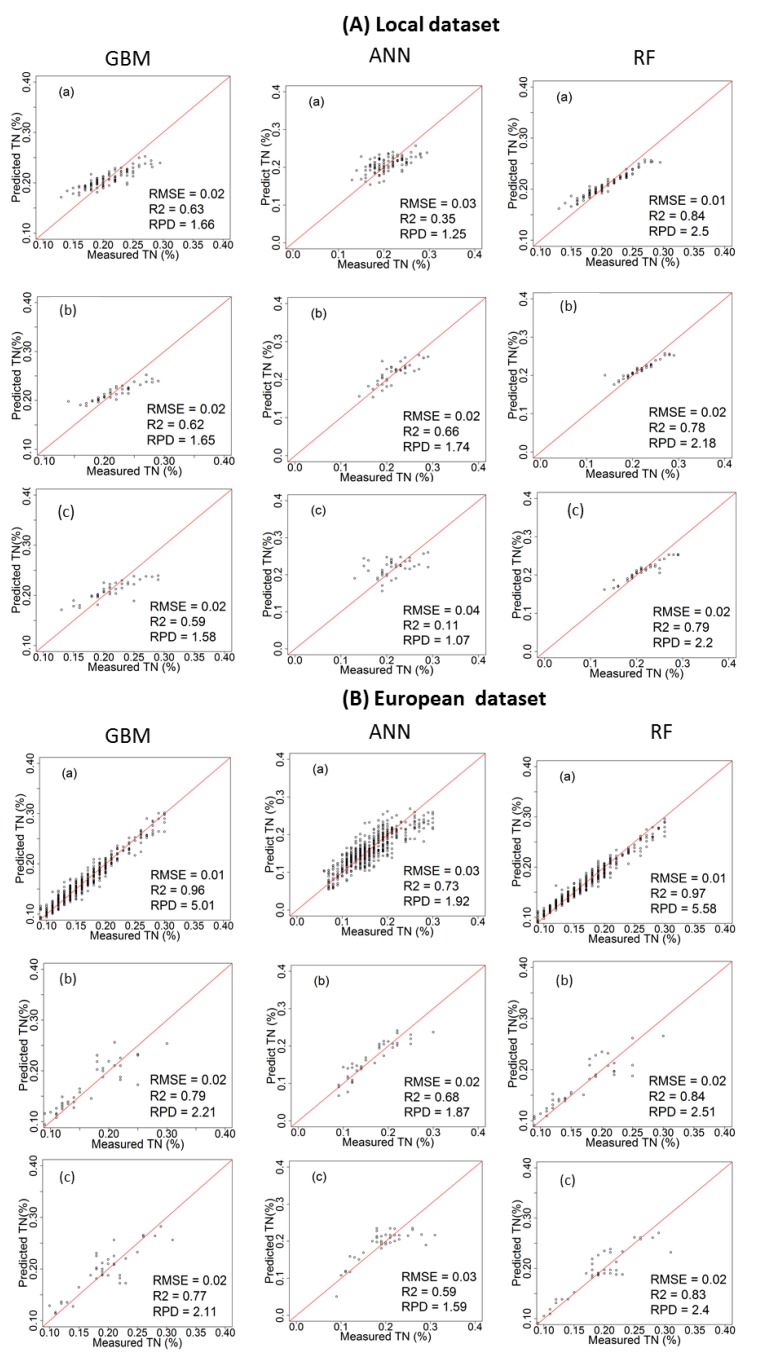
Scatter plots of visible and near infrared (vis-NIR)-predicted versus laboratory-analysed total nitrogen (TN) in Hagg field in cross validation (**a**); lab prediction (**b**) and on-line prediction (**c**), using local dataset (**A**) and spiked European dataset (**B**); comparing between gradient boosted machines (GBM); artificial neural network (ANNs) and random forests (RF) models.

Table 2 and Figure 4 and Figure 5 show the cross-validation, laboratory and on-line prediction results for TN calibration models developed with local and European datasets. In cross-validation, RF outperformed both GBM and ANN, successively, for modelling TN. The best results achieved with RF are based on the spiked European dataset with R^2^ = 0.97, RMSECV = 0.01%, and RPD = 5.58 for the Hagg field, and R^2^ = 0.96, RMSECV = 0.01%, and RPD = 4.83 for the Hessleskew field (Table 2). The lowest results are obtained with ANN based on local dataset with R^2^ = 0.35, RMSE = 0.03%, and RPD = 1.25 for the Hagg field, and R^2^ = 0.62, RMSE = 0.01%, and RPD = 1.62 for the Hessleskew field.

The performance of the laboratory prediction shows a different trend to that of the cross-validation, where the GBM based on the spiked European dataset models generally provide the best results with R^2^ = 0.87, RMSE = 0.02%, and RPD = 2.79 in the Hesselskew field (Table 2; Figure 4), followed by RF model based on the spiked European dataset with R^2^ = 0.84, RMSE = 0.02%, and RPD = 2.51 in the Hagg field (Table 2; Figure 5). However, GBM-local dataset-based models in particular for the Hessleskew field has resulted in the least significant results (R^2^ = 0.60, RMSE = 0.01%, and RPD = 1.60), and R^2^ = 0.62, RMSE = 0.02%, and RPD = 1.65 in the Hagg field, shown in Table 2). ANN outperforms GBM in modeling based on local dataset with R^2^ of 0.69 and 0.66, RMSE of 0.01% and 0.02%, and RPD of 1.81 and 1.74 for the Hessleskew and Hagg field, respectively. However, RF local model shows better performances for laboratory prediction of TN, compared to both ANN and GMB based on the corresponding datasets, particularly in the Hesselskew field.

Like for the cross-validation, the best results of on-line prediction are obtained with RF, followed successively by GBM and ANN (Table 2). This is true in the Hagg field, where the highest results of RF model obtained with the spiked European dataset with R^2^ = 0.83, RMSE = 0.02%, and RPD = 2.40, followed by RF model based on local dataset with R^2^ = 0.79, RMSE = 0.02%, and RPD = 2.20 in the Hagg field (Table 2; Figure 5). The local-ANN based model has the lowest results in the Hagg field (R^2^ = 0.11, RMSE = 0.04%, and RPD = 1.07) and the Hessleskew field (R^2^ = 0.26, RMSE = 0.02%, and RPD = 1.18), followed by GBM local model for the the Hessleskew field (R^2^ = 0.53, RMSE = 0.02%, and RPD = 1.48, Table 2). However, the model prediction performance varies between the two studied fields. Although the best performing local dataset was with RF, ANN provides a better prediction with the spiked dataset in the Hesselskew field, although the differences are small compared to RF and GBM.

### 3.3. Performance of the Calibration Models for Predicting TC

**Figure 6:**
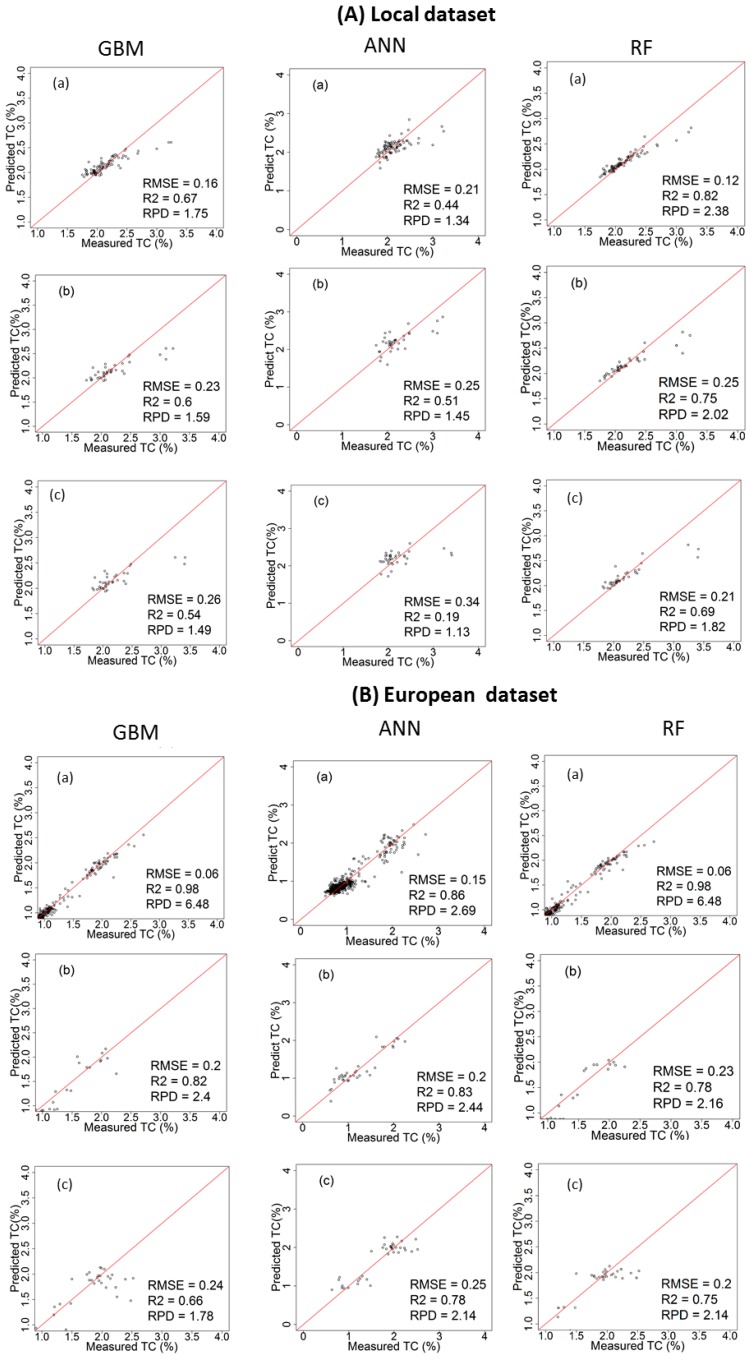
Scatter plots of visible and near infrared (vis-NIR)-predicted versus laboratory-analysed total carbon (TC) in the Hesselskew field in cross validation (**a**); lab prediction (**b**) and on-line prediction (**c**); using local dataset (**A**) and spiked European dataset (**B**); comparing between gradient boosted machines (GBM), artificial neural networks (ANNs) and random forests (RF) models.

**Figure 7:**
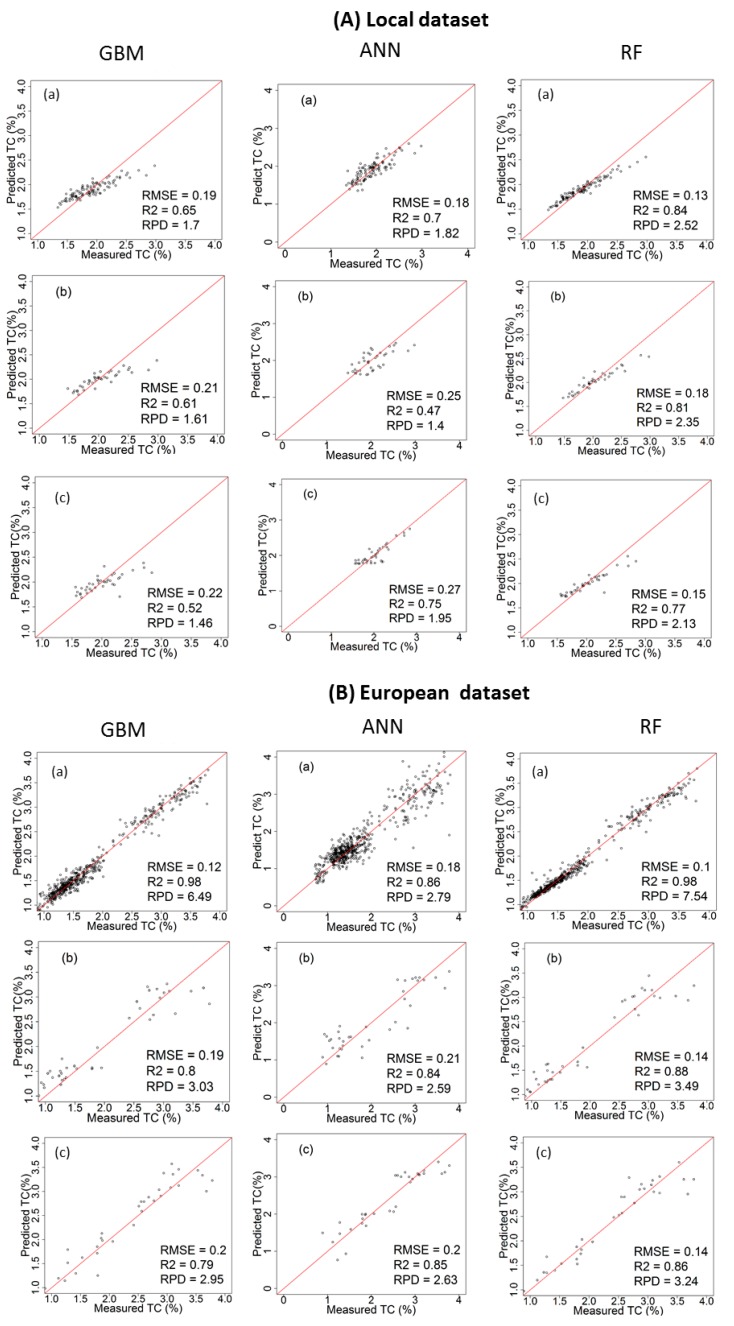
Scatter plots of visible and near infrared (vis-NIR)-predicted versus laboratory-analysed total carbon (TC) in the Hagg field in cross validation (**a**); lab prediction (**b**) and on-line prediction (**c**); using local dataset (**A**) and spiked European dataset (**B**); comparing between gradient boosted machines (GBM), artificial neural networks (ANNs) and random forests (RF) models.

Table 2 and Figure 6 and Figure 7 show the results of cross-validation, laboratory and on-line predictions. For cross-validation, the RF outperformed both GBM and ANN models. The best results are achieved with RF using the spiked European dataset in both fields with R^2^ = 0.98, RMSECV = 0.10% and RPD = 7.54 for the Hagg field, and R^2^ = 0.98, RMSECV = 0.06% and RPD = 6.48 for the Hesselskew field. However, the performance of the RF model in the Hessleskew field is identical to that of GBM. While the results of local-ANN based model is the poorest in the Hessleskew field with R^2^ = 0.44, RMSE = 0.21% and RPD = 1. 34, followed by local-GBM based model in the Hagg field with R^2^ = 0.65, RMSECV = 0.19% and RPD = 1.70. Overall, the cross-validation results for TC is identical to that for TN reported above.

**Figure 8:**
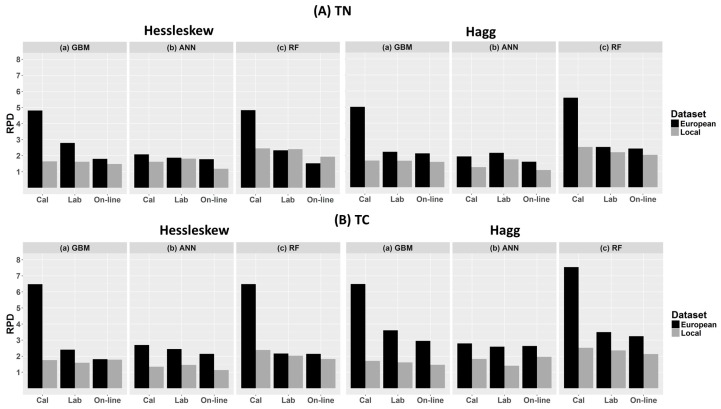
Comparison of residual prediction deviation (RPD) values for (**A**) total nitrogen (TN) and (**B**) total carbon (TC) predictions obtained with (**a**) gradient boosted machines (GBM); (**b**) artificial neural networks (ANNs) and (**c**) random forests (RF) analyses in cross-validation (Cal), laboratory prediction (Lab) and on-line prediction (Online). Results were generated with local field datasets of 122 and 149 samples for the Hessleskew and Hagg fields, respectively, and a spiked European dataset (528 samples).

The laboratory prediction of TC behaves similarly to the cross-validation stage, where RF over-performs both GBM and ANN (Table 2), with the best results obtained for the RF model based on the spiked European dataset (R^2^ = 0.88, RMSE = 0. 14% and RPD = 3.49 in the Hagg field, followed by GBM model based on the spiked European dataset (R^2^ = 0.83, RMSE = 0. 15% and RPD = 3.16 in the Hagg field). However, ANN model outperforms both RF and GBM in the Hessleskew field with the spiked European dataset (R^2^ = 0.83, RMSE = 0.20% and RPD = 2.44). RF models outperform both GBM and ANN models based on the local dataset in both Hesselsekew and Hagg fields (Table 2 and Figure 8).

Similarly to the laboratory prediction, the best results for the on-line prediction are achieved using RF based on the spiked European dataset in the Hagg field (R^2^ = 0.86, RMSE = 0.14% and RPD = 3.24), followed by GBM based on the spiked European dataset in the Hagg field also (R^2^ = 0.85, RMSE = 0. 20% and RPD = 2.95). Again, ANN based on the spiked European dataset outperform both RF and GBM in the Hessleskew field (R^2^ = 0.78, RMSE = 0.25% and RPD = 2.14). For the local dataset based models, RF outperforms both GBM and ANN models in both the Hagg and Hessleskew fields (Table 2; Figure 8).

## 4. Discussion

### 4.1. Comparison of Model Performance

**Figure 9:**
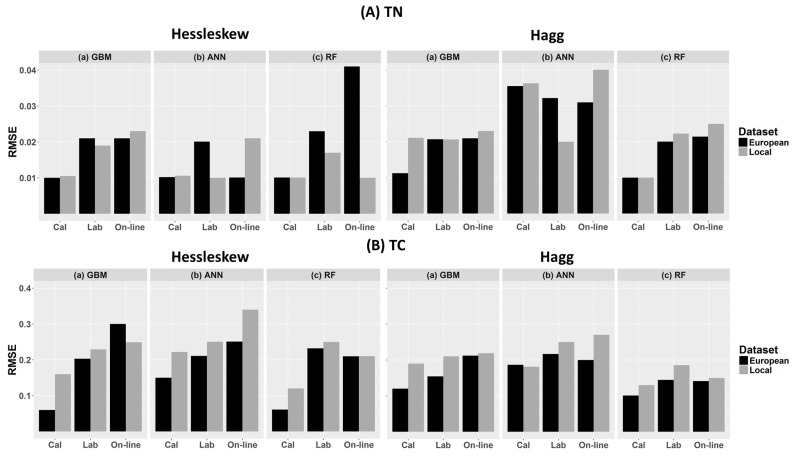
Comparison of root mean square error (RMSE) values for (**A**) total nitrogen (TN) and (**B**) total carbon (TC) predictions obtained with (**a**) gradient boosted machines (GBM); (**b**) artificial neural networks (ANNs) and (**c**) random forests (RF) analyses in cross-validation (Cal), laboratory prediction (Lab) and on-line prediction (Online). Results were generated with local field datasets of 122 and 149 samples for the Hessleskew and Hagg fields, respectively, and a spiked European dataset (528 samples).

In this work we compared the accuracy of the GBM, ANN and RF methods for the prediction of TN and TC based on local and European datasets. The variations of R^2^, RPD as well as RMSE values obtained from cross-validation, laboratory and on-line prediction are shown in Table 2 and Figure 8 and Figure 9.

Although RF models have resulted in the highest prediction performance followed by GBM in cross-validation, this was the case for the laboratory and on-line predictions in the Hagg field only, whereas ANN models based on the spiked European dataset has provided improved results for the laboratory (for TC only) and on-line (for both TC and TN) predictions in the Hessleskew field only. This means that the laboratory prediction followed the same trend as for cross-validation in the Hagg field only, where RF outperform both GBM and ANN. Sorenson et al. [15] found RF to outperform ANN for the prediction of OC and TN for non-mobile measurement, reporting RMSE = 0.62 and 1.56%, and RPD = 2.1 and 0.90 for RF and ANN, respectively, for OC and RMSE of 0.60 and 0.12%, and RPD of 2.1 and 1.0 for RF and ANN, respectively for TN. Viscarra Rossel and Behrens [14] reported better prediction results for RF compared to BT, but was less performing than ANN using the discrete wavelet transform as the predictors (RMSEP of 0.99%, 0.93% and 0.75%, and R^2^ of 0.83, 0.84, and 0.89 for DWT-BT, DWT-RF, and DWT-ANN, respectively). This points out that, depending on the geographic region, one method may outperform several others [47].

Similar to the on-line predictions, the results of RF for the Hagg field are better than those in the Hessleskew field, which is in line with the results reported by Nawar and Mouazen [5] for on-line measurement based on the spiked European dataset (RMSEP of 0.03–0.19% and RPD of 5.21–5.94 for TN and TC, respectively). However, results in this research are better than those reported by Kuang and Mouazen [48] using PLSR, with RPD of 2.52 and 2.33 for TN and OC, respectively and better than ANN models [11] for on-line prediction of OC (RPD = 2.28, compared to RPD of 2.52 of the current research). RF outperforms both GBM and ANN for on-line predictions in the Hagg field only, whereas ANN replaces RF in being the best performing for on-line prediction in the Hessleskew field. From the quality of on-line prediction of the studied two soil properties it can be concluded that ANN might perform equally as RF, which rejects the hypothesis of the current work that both RF and GBM outperform ANN for on-line prediction of TN and TC.

### 4.2. Influence of Dataset on Models’ Performance

The influence of dataset size and concentration range showed great influences on the performance in calibration and prediction. The results associated with spiking local samples into the European dataset more often enhances the overall model performance, especially for cross-validation, in comparison with those obtained using the local dataset (Table 2 and Figure 8), which is in agreement with the results presented by Brown [49] and Sankey et al. [50] for non-mobile measurements, and Kuang and Mouazen [48] for on-line measurements. The improvement was mainly expressed as improved R^2^ and RPD, and RMSEP in laboratory and on-line predictions (Table 2 and Figure 9). This finding is in agreement with Kuang and Mouazen [48], who reported improvement in R^2^ and RPD for predictions of TN and TC by adding local samples into a general library. Furthermore, Nawar and Mouazen [5] reported that the spiking of local soil samples into European datasets turned out to be a competent method to enhance the prediction associated with target field samples. Compared to published results, using the spiking of target field samples into European samples obtained with PLSR analyses [48] for TN (RPD = 1.96–2.52) and OC (RPD = 1.88–2.38), the results of on-line prediction based on RF in the current research is better for TN and TC, and better than those results reported for on-line measurement of OC by Kuang et al. [11] using ANN analysis with RPD and RMSE values of 2.28 and 1.25%, respectively. Taking into account the small variation range of TN and TC in the two scanned fields (Table 1), spiking of the European dataset with local samples seems to provide the best scenario to improve on-line prediction performance. This was also proved to be true for laboratory-scanned (non-mobile) soil spectra spiked into global or European datasets [51,52].

A possible explanation for the high performance of both the laboratory and on-line predictions with the spiked European data set is the wider concentration ranges (larger variability) within the datasets for both properties compared to the narrow range of the local datasets. This wide range or variability is indeed a fundamental factor in the calibration of the vis-NIR spectroscopy which is essential for successful modelling of data, particularly in fields with narrow concentration ranges. In fact, if the concentration range in a field is too narrow, no calibration models can be established at all, and it will be essential at this point to spike selected samples from a target field into existing spectral library with wide concentration range. This implies that the overall model performance may depend to a large extent of variability exist in the dataset [53]. This is the reason why researchers have concluded that calibration models should be established based on libraries that capture wide concentration range and soil types [54]. Kuang and Mouazen [6] reported that fields with small variations in concentrations of a given soil property will properly lead to inferior model performance (small R^2^ and RPD). Furthermore, Nawar and Mouazen [5] found that spiked local sample with small variation (small range) into an European dataset with wide concentration range improved the on-line prediction (in terms of improved R^2^ and RPD and decreased RMSEP) of TN and TC compared to local datasets.

## 5. Conclusions

In this study the performance of generalized boosted machines (GBM), artificial neural networks (ANNs), and random forests (RF) methods was compared for the visible and near infrared spectroscopy prediction of soil total nitrogen (TN) and total carbon (TC) under laboratory (non-mobile) and on-line (mobile) scanning conditions in two selected fields in the UK (Hessleskew and Hagg fields). We have tested the performance of these modelling methods using local and European datasets, spiked with samples from the two target fields. Generally, the performance of the GBM, ANN and RF models varied according to the dataset used. Results showed the majority of the RF models to outperform the corresponding GBM and ANN models in cross-validation, laboratory and on-line predictions. Results in cross-validation showed improved performance with the spiked European dataset that were collected from 16 fields in five European countries. Nevertheless, the performance of laboratory and on-line predictions does not necessarily behave similarly to cross-validation. The ANN model based on the spiked European dataset showed better performance than RF and GBM in laboratory (for TC only) and on-line prediction (for TC and TN) in the Hessleskew field only. The highest on-line prediction results were observed with RF models in the Hagg field based on the spiked European dataset.

From the results obtained in this work, it is observed that calibrations obtained with the spiked European dataset is the most successful option for on-line predictions of the TN and TC, compared to field local calibration. The spiked European calibrations based on 528 samples provided a larger coefficient of determination (R^2^) and residual prediction deviation (RPD) compared to the local calibration models for TN and TC in both fields. Future work needs to focus on optimizing the selection of an optimal dataset to be spiked into the European dataset. This needs to test distance matrices and sample selection algorithms for potential improvement in the prediction quality of resulted models compared to random sample selection based modelling.

## Figures and Tables

**Table 1:** Descriptive statistics for soil total carbon (TC) and total nitrogen (TN) for Hessleskew, Hagg fields, and European datasets.

	Min	1st Qu.	Median	Mean	3rd Qu.	Max	St.dev
**Hessleskew**	**(*n* = 122)**						
TN (%)	0.19	0.23	0.25	0.25	0.26	0.34	0.02
TC (%)	1.72	1.94	2.05	2.12	2.22	3.67	0.30
**Hagg**	**(*n* = 149)**						
TN (%)	0.13	0.19	0.21	0.21	0.24	0.35	0.04
TC (%)	1.34	1.68	1.90	1.92	2.08	3.18	0.31
**European**	**(*n* = 528)**						
TN (%)	0.03	0.11	0.14	0.15	0.17	0.30	0.04
TC (%)	0.45	1.22	1.45	1.67	1.70	3.76	0.77

**Table 2:** Hessleskew and Hagg fields results in cross-validation, laboratory and on-line predictions using local, and spiked European dataset based on gradient boosted machines (GBM), artificial neural networks (ANNs) and random forests (RF) models.

Hessleskew									Hagg							
			Local			European					Local			European		
			RMSE	R^2^	RPD	RMSE	R^2^	RPD			RMSE	R^2^	RPD	RMSE	R^2^	RPD
**GBM**	n.trees								n.trees							
Cross-	100	TN	0.01	0.63	1.64	0.01	0.96	4.81	100	TN	0.02	0.63	1.66	0.01	0.96	5.01
	100	TC	0.16	0.67	1.75	0.06	0.98	6.48	100	TC	0.19	0.65	1.70	0.12	0.98	6.49
Lab Prediction	100	TN	0.01	0.60	1.60	0.02	0.87	2.79	100	TN	0.02	0.62	1.65	0.02	0.79	2.21
	100	TC	0.23	0.60	1.59	0.20	0.82	2.40	100	TC	0.21	0.61	1.61	0.19	0.83	3.03
On-line Prediction	100	TN	0.02	0.53	1.48	0.02	0.66	1.80	100	TN	0.02	0.59	1.58	0.02	0.77	2.11
	100	TC	0.26	0.54	1.49	0.24	0.66	1.78	100	TC	0.22	0.52	1.46	0.20	0.79	2.95
**ANN**	size								size							
Cross- validation	2	TN	0.01	0.62	1.62	0.01	0.77	2.08	2	TN	0.03	0.35	1.25	0.03	0.73	1.92
	2	TC	0.21	0.44	1.34	0.15	0.86	2.69	2	TC	0.18	0.70	1.82	0.18	0.86	2.79
Lab Prediction	2	TN	0.01	0.69	1.81	0.01	0.71	2.02	2	TN	0.02	0.66	1.74	0.02	0.68	1.87
	2	TC	0.25	0.51	1.45	0.20	0.83	2.44	2	TC	0.25	0.47	1.40	0.21	0.84	2.59
On-line Prediction	2	TN	0.02	0.26	1.18	0.01	0.68	1.78	2	TN	0.04	0.11	1.07	0.03	0.59	1.59
	2	TC	0.34	0.19	1.13	0.25	0.78	2.14	2	TC	0.27	0.75	1. 95	0.20	0.85	2.63
**RF**	ntree								ntree							
Cross- validation	100	TN	0.01	0.83	2.45	0.01	0.96	4.83	100	TN	0.01	0.84	2.50	0.01	0.97	5.58
	100	TC	0.12	0.82	2.38	0.06	0.98	6.48	100	TC	0.13	0.84	2.52	0.10	0.98	7.54
Lab Prediction	100	TN	0.01	0.82	2.40	0.02	0.81	2.33	100	TN	0.02	0.78	2.18	0.02	0.84	2.51
	100	TC	0.25	0.75	2.02	0.23	0.78	2.16	100	TC	0.18	0.81	2.35	0.14	0.88	3.49
On-line Prediction	100	TN	0.01	0.72	1.93	0.04	0.55	1.52	100	TN	0.02	0.79	2. 20	0.02	0.83	2.40
	100	TC	0.21	0.69	1.82	0.20	0.75	2.13	100	TC	0.15	0.77	2.13	0.14	0.86	3.24

n.trees = total number of trees to fit., size = number of units in the hidden layer, ntree = number of trees.
